# Can different scores in first and second halves influence running and explosive-based measures?

**DOI:** 10.5114/biolsport.2025.144296

**Published:** 2024-10-23

**Authors:** Ryland Morgans, John Radnor, Jon Oliver, Jule Scholten, Piotr Zmijewski, Ronan Kavanagh, Ben Ryan, Chris Haslam, Matthew King, Rafael Oliveira

**Affiliations:** 1 School of Sport and Health Sciences, Cardiff Metropolitan University, Cardiff, UK; 2 Brentford FC Football Research Centre, Brentford FC, London, UK; 3 Jozef Pilsudski University of Physical Education in Warsaw, 00-809 Warsaw, Poland; 4 Football Performance Hub, School of Sport and Health Sciences, University of Central Lancashire, Preston PR1 2HE, UK; 5 Research Centre in Sports Sciences, Health and Human Development (CIDESD), Santarém Polytechnic University, 2040-413 Rio Maior, Portugal; 6 Santarém Polytechnic University, School of Sport, 2040-413 Rio Maior, Portugal

**Keywords:** External load, Contextual variables, Match result, Match half, Football

## Abstract

This study aimed to examine the influence of different scores in the first and second half on running and explosive-based performance of elite male soccer players. Thirty-three professional players from one English Premier League team participated in the study across two consecutive seasons, 2021/22 and 2022/23. Matches were divided into half (first versus second) and nine phases; WIN-WIN; WIN-DRAW; WIN-LOSS; DRAW-WIN; DRAW-DRAW; DRAW-LOSS; LOSS-WIN; LOSS-DRAW; and LOSS-LOSS. Match physical data were monitored using an 18 Hz Global Positioning System. There was a main effect for half for all variables (*p* < 0.001–0.008; η^2^ = 0.004–0.028), with distances covered per minute and number of explosive actions per minute greater in the first-half than second-half (*d* = 0.144–0.374). There was an interaction effect between half and phase for m/min, high-speed running per min, high metabolic load distance (HMLD) per min, HML efforts/min, and accelerations/min (*p* < 0.001–0.012; η^2^ = 0.010–0.015). There was a reduction between first-half and second-half performance during WIN-WIN, WIN-DRAW, DRAW-WIN, DRAW-DRAW, LOSE-WIN, and LOSE-LOSE for m/min (*p* < 0.001; *d* = 0.435–0.714), HMLD/min (*p* < 0.001–0.004; *d* = 0.334–0.605), and HML efforts/min (*p* < 0.001; *d* = 0.408–0.611). In conclusion, our findings emphasise the importance of considering both match half and phase when analysing players’ physical performance to support the prescription of tailored training programs and tactical strategies to optimise performance across different match situations.

## INTRODUCTION

The main aim of a soccer match is to score more goals than the opponent in order to have a successful result and win the match [1]. Thus, the final match result (win, draw or lose) may be the most relevant contextual/situational variable to determine success [2–4]. The physical variables related to sprinting, accelerating and decelerating are usually associated with specific soccer match-play actions, such as, scoring a goal [6]. Previously, it was shown that accelerations achieve 7–10% and decelerations 57% of the total player load across various playing positions in Norwegian professional soccer players during match-play [7].

Another relevant variable is the high metabolic load distance (HMLD) which refers to the distance covered with a power consumption above 25.5 W/kg. This value corresponds to running at a constant velocity of 5.5 m/s or 19.8 km/h on grass [8] and it also includes accelerations and decelerations (e.g., from 2–4 m · s^−2^ for 1 s). Altogether, HMLD provides information about high-intensity actions as it includes high-speed running, accelerations and decelerations, which justifies its analysis in soccer.

When considering the final match result to determine the success of a team, a study found that soccer players covered more distance across all running intensities while winning [3]. However, the result of the match is not the only contextual variable to influence running or explosive-based variables. For instance, match half, first- or second-half, can influence acceleration and decelerations [8]. A study found higher acceleration and deceleration values in the first-half compared with the second-half. Additionally, higher values occurred during positive match results (win and draw) compared with negative outcomes (loss) [8]. However, this study only considered the final match result for the comparisons which does account for changes in score-line during the match. Furthermore, several studies found differences between match halves, reporting higher values in the first-half compared with the second-half [3, 8–10]. Additionally, some research has analysed acceleration and deceleration differences in relation to the final match result [3, 8, 9]. For example, higher values in the first-half were found regardless of the final match result [3, 8]. Even so, the previous research did not include the different phase analysis when changing from the first-half to the second-half of the match.

Considering the various phase analysis, recent research examined seven phases of match-play [4, 5]. Specifically, these seven phases of match-play were related to scoring, drawing or losing. The seven phases included: 1) from drawing to winning (DW), 2) from losing to drawing (LD), 3) continuation of winning (WW), 4) drawing (DD), 5) losing (LL), 6) from drawing to losing (DL) and lastly, 7) from winning to drawing (WD) [4, 5].

Earlier research examined technical activity and found that to keep a winning status, a higher frequency of shot occurrence, particularly on target and a higher frequency and effectiveness of dribbling are needed [5]. While more recently, Konefal et al. [4] analysed total distance and high-intensity running distance (> 5.5 m/s) during various match phases and observed that higher total distance was performed during DW, DL and DD phases when compared with the other phases. Moreover, greater values of high-intensity running distance were recorded during DW, DL and LL phases. Finally, the study also noted that the lowest total distance and high-intensity distance was covered during the WD phase. However, the study did not include other explosive type variables (e.g., accelerations or decelerations) and to the best of the present authors knowledge, there is no other research with a similar approach or utilising explosive type variables.

Therefore, the purpose of this research was to examine the influence of different scores in the first and second half on running and explosive-based performance of elite male soccer players, during two consecutive seasons. As such, all possible results that changed from the first- to the second-half of each match were considered. Considering recent research [4], it was hypothesised that running and explosive-based performance actions of elite soccer players will be different across the nine phases of match status in the English Premier League (EPL).

## MATERIALS AND METHODS

### Participants

This observational study involved professional soccer players from one EPL team across two consecutive seasons, 2021/22 and 2022/23. Thirty-three first-team squad players (age 23.2 ± 5.9 years, weight 75.2 ± 8.1 kg, height 1.83 ± 0.06 m) participated in the study. Data was obtained from all official matches played during both seasons (EPL n = 38 in each season). Only outfield players who completed the entire match (+90 min) were included for analysis. Goalkeepers were excluded from the investigation due to the specific nature of the match activity and low running demands [11]. All data resulted from normal analytical procedures regarding player monitoring over the competitive season, nevertheless, written informed consent was obtained from all participants. To ensure the anonymity of the players all data were anonymised prior to analysis in accordance with the Declaration of Helsinki. Moreover, this study was approved by Cardiff Metropolitan University local ethics committee and the professional club from which the participants volunteered [12].

### Data Collection Procedures

Match status was divided into nine phases that resulted in changing or maintaining the match outcome adapted from existing research [4, 5]; WIN-WIN, when the winning team maintains the winning status of the match; WIN-DRAW, when the winning team concede a goal and the match status changes to drawing; WIN-LOSS, when the winning team concede a goal and the match status changes to lose; DRAW-WIN, when one of the drawing teams score a goal and changes the status to winning; DRAW-DRAW, when the drawing teams maintains this status; DRAW-LOSS, when a drawing team concede a goal and the match status changes to losing; LOSS-WIN, when the losing team score a goal and the match status changes to winning; LOSS-DRAW, when the losing team score a goal and the match status changes to drawing; and LOSS-LOSS, when the losing team maintains the losing status. From the two seasons, the number for each phase were; WIN-WIN = 84, WIN-DRAW = 76, WIN-LOSS = 29, DRAW-WIN = 68, DRAW-DRAW = 153, DRAW-LOSS = 47, LOSS-WIN = 89, LOSS-DRAW = 84, LOSS-LOSS = 83. The league status was determined and recorded by the lead researcher and based on the current league standard of competition.

Match running and accelerating and decelerating data were consistently monitored across the study seasons using an 18 Hz Global Positioning System (GPS) technology tracking system (Apex Pod, version 4.03, 50 gr, 88 × 33 mm; Statsports; Northern Ireland, UK) that has been previously validated for tracking distance covered and peak velocity during simulated team sports and linear sprinting [13]. All data collection procedures and unit error and reliability have previously been reported [13–16]. The distance biases for the Apex 18 Hz during a 400 m trial (1.17 ± 0.73%), 128.5 m circuit (2.11 ± 1.06%), and 20 m trial (1.15 ± 1.23%) have previously been reported [13], where these units reported a small error of around 1–2% compared to criterion distances and good levels of accuracy (bias < 5%) in sport specific metrics [13]. All devices were activated 30-minutes before data collection to allow the acquisition of satellite signals and to synchronise the GPS clock with the satellite’s atomic clock [17]. To avoid potential inter-unit variation players wore the same GPS unit for each training session and match [16], although the present GPS system has previously reported excellent inter-unit reliability [18]. Specifically designed vests were used to hold the devices, located on the player’s upper torso, and anatomically adjusted to each player, as previously described [16]. The GPS signal quality and horizontal dilution of position were connected to a mean number of 21 ± 3 satellites, range 18–23, while HDOP for all seasons ranged between 0.9–1.3.

On completion of each match, GPS data were extracted using proprietary software (Apex, 10 Hz version 4.3.8, Statsports Software; Northern Ireland, UK) as software-derived data is a more simple and efficient way for practitioners to obtain data in an applied environment, with no differences reported between processing methods (software-derived to raw processed) [19]. The dwell time (minimum effort duration) was set at 0.5 s to detect high-intensity running and 1 s to detect sprint distance efforts, in-line with manufacturers recommendations and default settings to maintain consistent data processing [19]. Furthermore, the internal processing of the GPS units utilised the Doppler shift method to calculate both distance and velocity data which is shown to display a higher level of precision and less error compared with data calculated via positional differentiation [20].

Variables analysed were selected based on previous literature [21, 22–24] and in practical settings are commonly utilised by elite soccer practitioners. Relative distances covered per minute (m/min) in the following categories were used for analysis: total distance covered (m/min), high-speed running distance (HSR) (m/min; 5.5–7 m/s), sprint distance (m/min; distance covered > 7 m/s), high metabolic load distance (HMLD) (m/min; the total amount of HSR, coupled with the total distance of accelerations (> 3 m/s^2^) and decelerations (< -3 m/s^2^)) [25]. The number of high metabolic (HML) efforts (n/min), number of sprint efforts (n/min), and accelerations and decelerations (n/min; > 3 m/s^2^) were also examined.

### Statistical analysis

Descriptive data (mean ± *SD*) were determined for all GPS variables of interest for each half of each phase (Phase 1: WIN-WIN; Phase 2: WIN-DRAW; Phase 3: WIN-LOSS; Phase 4: DRAW-WIN; Phase 5: DRAW-DRAW; Phase 6: DRAW-LOSS; Phase 7: LOSS-WIN; Phase 8: LOSS-DRAW; Phase 9: LOSS-LOSS). Homogeneity of variance was assessed via Levene’s statistic and, where violated, Welch’s adjustment was used to correct the F-ratio. To investigate differences in match running performance between the different phases and halves, multiple two-way (9 × 2) ANOVA were conducted with the nine phases as the between-subjects variable and match half as the within-subject variable. Post-hoc analysis, using either Bonferroni or Games-Howell post-hoc analyses, where equal variances were and were not assumed, was conducted to identify the differences in match running demands between halves for each phase. Multiple one-way ANOVA was conducted to explore the difference between first-half and second-half performance across phases of the same half-time outcome.

Effect size (*η*^2^) values were reported for the ANOVA results, while Cohen’s *d* values (*d*) were reported for significant results. Eta square partial (*η*^2^) values in the range 0–0.0099 are considered insignificant effect sizes, 0.0100–0.0588 as small effect sizes, 0.0589–0.1379 as medium effect sizes, and values greater than 0.1379 as large effect sizes [26]. Cohens *d* effect size magnitudes were interpreted using the following classifications: trivial < 0.19; small 0.2–0.59; 0.6–1.19 moderate; 1.2–1.9 large; 2.0–3.9 very large; > 4.0 extremely large [27]. All significance values were accepted at *p* < 0.05 and all statistical procedures were conducted using JASP (version 0.18) for Macintosh.

## RESULTS

**FIG. 1:**
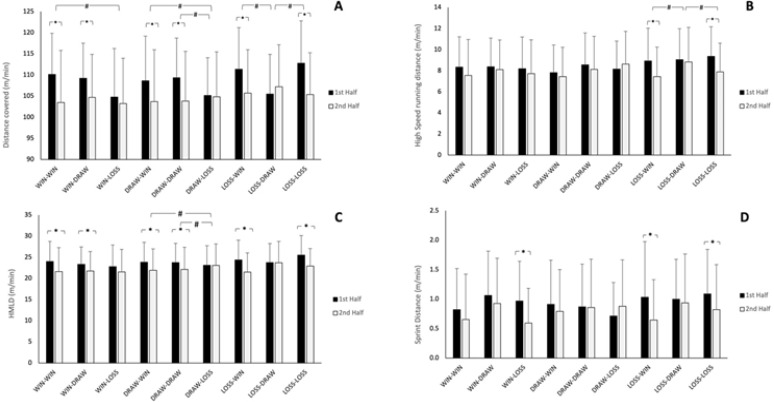
Descriptive statistics (mean ± SD) for first half and second half running performance across all stages. A: Distance Covered (m/min); B: High Speed Running Distance (m/min); C: HMLD (m/min; D: Sprint Distance (m/min). * Indicates significant difference (p < 0.05), HMLD: high metabolic load distance

**FIG. 2:**
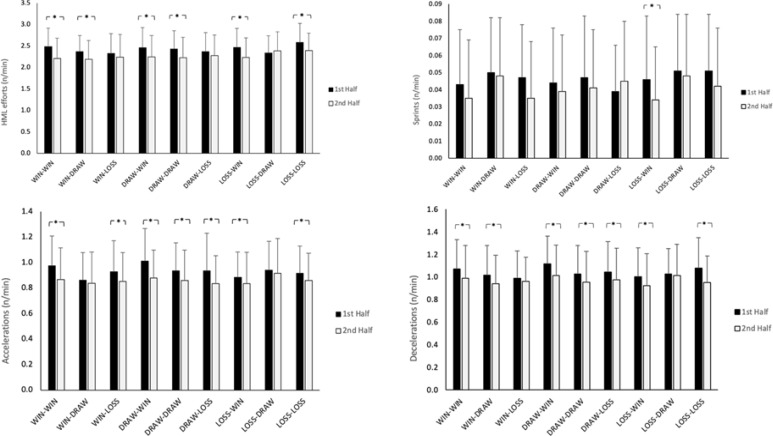
Descriptive statistics (mean ± SD) for first half and second half explosive actions across all stages. * Indicates significant difference (p < 0.05), HML: high metabolic load

Descriptive statistics (mean ± standard deviation) for distances covered and number of explosive efforts for the various phases and for each half are presented in Figure 1 and Figure 2. There was a significant main effect of half for all variables (*p* < 0.001–0.008; *η*^2^ = 0.004–0.028), with distances covered per minute and number of explosive actions per minute greater in the first-half than second-half (*d* = 0.144–0.374). However, there was no main effects for phase (*p* = 0.060–0.260; *η*^2^ = 0.010–0.015) for any of the variables.

There were an interaction effects between phase and half for m/min, HSR/min, HMLD/min, HML efforts/min, and accelerations/min (*p* < 0.001–0.012; *η*^2^ = 0.010–0.015). There was a reduction between first-half and second-half performance for the following phases; WIN-WIN, WIN-DRAW, DRAW-WIN, DRAW-DRAW, LOSE-WIN, and LOSE-LOSE for m/min (*p* < 0.001; *d* = 0.435–0.714), HMLD/min (*p* < 0.001–0.004; *d* = 0.334–0.605), and HML efforts/min (*p* < 0.001; *d* = 0.408–0.611). For HSR/min, there was lower running performance in the second-half compared to the first-half for LOSE-WIN and LOSE-LOSE (*p* < 0.001; *d* = 0.498–0.506). In terms of accelerations/min, there were less accelerations in the second-half compared to the first-half for WIN-WIN, DRAW-WIN, and DRAW-DRAW (*p* < 0.001; *d* = 0.319–0.567).

The interaction between half and phase also revealed greater distance covered per minute in the first-half of LOSS-LOSS compared to DRAW-LOSS, and LOSS-DRAW (*p* = 0.001–0.009; *d* = 0.704–0.738), while also greater distances covered per-minute in the first-half of LOSS-WIN compared to LOSS-DRAW (*p* = 0.030; *d* = 0.568). There were also more accelerations per minute in the first-half of DRAW-WIN compared to WIN-DRAW (*p* = 0.017; *d* = 0.647). There were no other differences between phases for within half performance.

Results from the separate one-way ANOVA comparing the difference in first- and second-half performance between phases are shown in Table 1. For relative distance covered (m/min) there was a difference between WIN-WIN and WIN-LOSS (*p* = 0.040; *d* = 0.545) for the difference between first-half and second-half performance. There was also a difference between DRAW-LOSS and both DRAW-WIN (*p* = 0.016; *d* = 0.532) and DRAW-DRAW (*p* = 0.001; *d* = 0.596) for the difference in relative distance covered between halves. Finally, there was also a difference between LOSS-DRAW and both LOSS-WIN (*p* < 0.001; *d* = 1.028) and LOSS-LOSS (*p* < 0.001; *d* = 1.265) for the difference between first-half and second-half relative distance covered.

**TABLE 1:** Difference between first half and second half performance for each stage for all GPS metrics

Stage	Relative Distance (m/min)	Relative HSR Distance (m/min)	Relative HMLD (m/min)	Relative Sprint Distance (m/min)	Relative HML efforts (n/min)	Relative Sprints (n/min)	Relative Accelerations (n/min)	Relative Decelerations (n/min)
WIN-WIN	-6.67 ± 9.63^a^	-0.79 ± 2.75	-2.39 ± 3.73	-0.17 ± 0.89	-0.27 ± 0.35^a^	-0.01 ± 0.04	-0.11 ± 0.23	-0.08 ± 0.22
WIN-DRAW	-4.50 ± 6.20	-0.27 ± 2.19	-1.59 ± 2.35	-0.13 ± 1.11	-0.18 ± 0.26	0.00 ± 0.05	-0.02 ± 0.17	-0.08 ± 0.23
WIN-LOSS	-1.55 ± 9.29	-0.46 ± 2.33	-1.24 ± 3.73	-0.38 ± 0.80	-0.08 ± 0.33	-0.01 ± 0.04	-0.08 ± 0.18	-0.03 ± 0.16
DRAW-WIN	-4.95 ± 9.61^b^	-0.39 ± 2.55	-1.91 ± 3.20	-0.12 ± 0.80	-0.21 ± 0.32	-0.01 ± 0.04	-0.13 ± 0.23	-0.10 ± 0.20
DRAW-DRAW	-5.50 ± 7.86^b^	-0.42 ± 2.56	-1.66 ± 2.97	-0.01 ± 0.90	-0.2 ± 0.32	-0.01 ± 0.05	-0.07 ± 0.19	-0.07 ± 0.21
DRAW-LOSS	-0.32 ± 9.79	0.46 ± 2.05	-0.05 ± 3.74	0.17 ± 1.10	-0.09 ± 0.37	0.01 ± 0.04	-0.10 ± 0.22	-0.07 ± 0.25
LOSS-WIN	-5.67 ± 7.75^c^	-1.50 ± 3.30^c^	-2.89 ± 3.86	-0.39 ± 1.16	-0.23 ± 0.34^c^	-0.01 ± 0.05	-0.05 ± 0.20	-0.08 ± 0.23
LOSS-DRAW	1.74 ± 6.67	-0.21 ± 2.64	-0.03 ± 2.92	-0.06 ± 1.07	0.05 ± 0.28	0.00 ± 0.05	-0.03 ± 0.23	-0.01 ± 0.22
LOSS-LOSS	-7.39 ± 7.22^c^	-1.47 ± 2.75^c^	-2.60 ± 3.16	-0.27 ± 1.02	-0.19 ± 0.31^c^	-0.01 ± 0.05	-0.05 ± 0.17	-0.13 ± 0.20^c^

**NOTE**: negative values indicate lower performance in second half;

a = significantly different to WIN-LOSS (*p* < 0.05);

b = significantly different to DRAW-LOSS (*p* < 0.05);

c = significantly different to LOSS-DRAW (*p* < 0.05);

HSR: High-speed running; HMLD: high metabolic load distance; HML: high metabolic load.

For relative HSR distance, there was a difference between LOSS-DRAW and both LOSS-WIN to (*p* = 0.012; *d* = 0.441) and LOSS-LOSS (*p* = 0.017; *d* = 0.432) for the difference in performance between halves. There was a difference between WIN-WIN and WIN-LOSS (*p* = 0.043; *d* = 0.539) for the difference in HML efforts between halves, as well as a difference between LOSS-DRAW and both LOSS-WIN (*p* < 0.001; *d* = 0.905) and LOSS-LOSS (*p* < 0.001; *d* = 0.767). Finally, for the number of decelerations per minute, there was a difference between LOSS-DRAW to LOSS-LOSS (*p* = 0.002; *d* = 0.538) for the difference in first-half to second-half performance.

## DISCUSSION

The main findings from the present study found a tendency of higher values of distances covered and explosive actions in the first-half than second-half with minor exceptions (LOSS-DRAW for total distance per minute, DRAW-LOSS for HSRD per minute, sprint per minute and number).

The decrease in distances covered and the number of explosive actions as the match progresses into the second-half is a phenomenon well-documented in soccer literature [3, 8–10]. Fatigue, both physical and mental, is often cited as a contributing factor to this decline [11, 16]. In addition, as players generally know the duration of the match, this can lead to different physical and tactical behaviours [28]. For instance, the initial time considered to complete different tasks usually present greater distances covered and at higher intensities than during later phases of the match due to specific pacing strategies of the players [28]. Moreover, strategic considerations, such as changes in tactics or conserving energy for critical moments, further contribute to the observed decline in players’ intensity levels during the latter phases of the match [11, 16]. Although, other contextual variables such as team formation, style of play, possession characteristics, match location, and opponent standard have recently been suggested to significantly contribute to the physical demands imposed on players [22, 29, 30]. This information can partly justify the previous exceptions (LOSS-DRAW for total distance per minute; DRAW-LOSS for HSRD per minute, number of sprints and sprint per minute). Thus, future studies should consider the previous contextual variables when analysing non-expected results.

Contrary to the main effect for half, the lack of a main effect for phase of match across any of the examined variables suggests that the observed differences in performance variables between the first and second halves are consistent throughout the match. This finding is supported by the notion that the decline in physical output and explosive actions is a continuous process rather than being influenced by specific match phases or events, as previously highlighted [19]. The results of the present study revealed an interaction effect between phase and half for multiple performance variables, with distances covered per minute and the number of explosive actions per minute being greater in the first-half compared to the second-half. Specifically, distance covered per minute (m/min), HSR/min, HMLD/min, HML efforts/min, and accelerations per minute. These findings suggest that the relationship between phase of match and half (time period within the match) significantly influences players’ physical outputs and movement patterns during a soccer match and are consistent with previous research [3, 8–10, 31]. This aligns with the notion that players are typically fresher and exert greater effort early in matches [31], since the knowledge of match duration and pacing strategies may contribute to effort regulation which consequently ensures players increase their exercise economy by improving positional relationships. As such, a possible consequence of this pacing strategy may be the diminishing focus on the distance between team-mates to allow the focus on other information to occur, such as ball location and the space available [28]. Furthermore, the importance of considering situational variables in understanding players’ physical demands during match-play and the influence on different playing positions have also been emphasised [22, 23, 30–33] and should be considered in future research.

Moreover, when comparing the differences between first-half and second-half for each phase, total distance, HSR, HML efforts and number of decelerations reported variations (see Table 1). Specifically, total distance and HML efforts revealed that when winning or drawing in the second-half, there was a greater difference from first- to second-half when compared with losing in the second-half (in all scenarios, such findings only occurred when winning in the first-half). This may partly be explained by the notion that when the team is losing, a higher running effort was made to achieve a better result (draw or win), which is ultimately the main aim of a soccer match [34]. This was also recently confirmed by another study that showed higher maximum velocity when the team was losing [32].

However, when examining HSR and HML efforts a different trend was reported, stating that when winning or losing in the second-half, a greater difference between the first- to second-half was observed compared with drawing in the second-half (in all scenarios, such findings only occurred when losing in the first-half). Finally, the difference in the number of decelerations was higher when losing in the first-half compared to the second-half and when losing to wining or drawing. In these specific cases, it would be expected that a similar scenario would be highlighted, although the lack of context concerning the level of opposition team [30], style of play and possession [22, 35] may justify such intriguing results [22, 23, 29–31, 33].

## Practical Applications, Limitations and Future Perspectives

Despite the interesting findings, this study presented some limitations. The sample derived from just one team with a specific context, country and league suggest that cautions must be considered when interpretating and generalising the results. However, the data included players that participated in the EPL across two seasons which is a strength of the current study. Factors such as individual player characteristics (e.g., playing position), match-specific variables (e.g., scoreline, playing style and formation), and environmental conditions (e.g., temperature, humidity) may also influence players’ performance throughout the match [29], although it is difficult to conduct research with a higher number of variables. Previous research has highlighted the importance of considering contextual factors in understanding match demands and player performance [36].

## CONCLUSIONS

In conclusion, the present study findings highlight the importance of monitoring players’ physical output and explosive actions throughout a match. Elite soccer players competing in the EPL, a tendency of greater distances covered and explosive actions in the first-half than second-half with minor exceptions (LOSS-DRAW for total distance, HML distance and efforts; DRAW-LOSS for HSR and sprint distances) were observed. Understanding these patterns can inform coaches and performance staff in optimising training, substitution strategies, and overall match preparation to maximise player performance across different phases of the match, as emphasised by earlier studies [4, 37]. Overall, our findings highlight the importance of considering both match phase and half when analysing players’ physical performance during soccer matches. By understanding how these factors interact to influence players’ movement patterns and intensity levels, coaches and performance staff can tailor training programs and tactical strategies to optimise performance across different match situations.
